# Synthesis and Evaluation of New Pyrazoline Derivatives as Potential Anticancer Agents

**DOI:** 10.3390/molecules201019066

**Published:** 2015-10-20

**Authors:** Muhammed Karabacak, Mehlika Dilek Altıntop, Halil İbrahim Çiftçi, Ryoko Koga, Masami Otsuka, Mikako Fujita, Ahmet Özdemir

**Affiliations:** 1 Department of Pharmaceutical Chemistry, Faculty of Pharmacy, Anadolu University, Eskişehir 26470, Turkey; E-Mails: rem1596@hotmail.com (M.K.); mdaltintop@anadolu.edu.tr (M.D.A.); 2 Department of Bioorganic Medicinal Chemistry, School of Pharmacy, Kumamoto University, Kumamoto 862-0973, Japan; E-Mails: 130y2011@st.kumamoto-u.ac.jp (H.İ.Ç.); kk1205@kumamoto-u.ac.jp (R.K.); motsuka@gpo.kumamoto-u.ac.jp (M.O.); 3 Research Institute for Drug Discovery, School of Pharmacy, Kumamoto University, Kumamoto 862-0973, Japan; E-Mail: mfujita@kumamoto-u.ac.jp

**Keywords:** pyrazoline, oxadiazole, anticancer activity, apoptosis, DNA cleavage

## Abstract

New pyrazoline derivatives were synthesized and evaluated for their cytotoxic effects on AsPC-1 human pancreatic adenocarcinoma, U87 and U251 human glioblastoma cell lines. 1-[((5-(4-Methylphenyl)-1,3,4-oxadiazol-2-yl)thio)acetyl]-3-(2-thienyl)-5-(4-chlorophenyl)-2-pyrazoline (**11**) was found to be the most effective anticancer agent against AsPC-1 and U251 cell lines, with IC_50_ values of 16.8 µM and 11.9 µM, respectively. Tumor selectivity of compound **11** was clearly seen between Jurkat human leukemic T-cell line and human peripheral blood mononuclear cells (PBMC). Due to its promising anticancer activity, compound **11** was chosen for apoptosis/necrosis evaluation and DNA-cleavage analysis in U251 cells. Compound **11**-treated U251 cells exhibited apoptotic phenotype at low concentration (1.5 µM). DNA-cleaving efficiency of this ligand was more significant than cisplatin and was clearly enhanced by Fe(II)-H_2_O_2_-ascorbic acid systems. This result pointed out the relationship between the DNA cleavage and the cell death.

## 1. Introduction

Cancer is not a single disease, but a large group of diseases characterized by uncontrolled, rapid, and pathological proliferation of abnormally transformed cells. Despite recent advances in cancer therapy, cancer is still the second leading cause of death after cardiovascular disorders throughout the world [1,2,3,4].

Resistance to chemotherapeutic agents remains a key challenge in the fight against cancer. Another challenge for chemotherapy is lack of selectivity. Generally anticancer drugs destroy normal cells as well as cancer cells and often cause serious adverse effects. Therefore, new antineoplastic agents are continually under development to selectively destroy tumour cells or at least limit their proliferation [1,2,3,4].

Diversely substituted pyrazolines embedded with a variety of functional groups are found in many important biologically-active compounds and considerable research on this class of agents has been carried out. They exhibit a wide spectrum of biological activities such as antimicrobial, anti-inflammatory, antidepressant, and anticancer effects. Among the reported activities, it is important to note that pyrazolines are not only useful in treatment of various cancer types, including brain, bone, mouth, esophagus, stomach, liver, bladder, pancreas, cervix, lung, breast, colon, rectum, and prostate cancers, but also some of them act as cancer chemopreventive agents [5,6,7,8,9,10,11,12,13,14,15,16,17,18,19,20,21,22,23]. In many studies, pyrazoline derivatives were reported as epidermal growth factor receptor tyrosine kinase (EGFR-TK) inhibitors [19], aurora kinase inhibitors [20], COX-2/B-Raf inhibitors [21], telomerase inhibitors [22], tubulin assembling inhibitors [23]. Additionally, 1,3,4-oxadiazole has emerged as an important scaffold owing to its metabolic profile and ability to engage in hydrogen bonding with receptor site. Recent studies have indicated that 1,3,4-oxadiazole derivatives exhibit potent anticancer activity against different cancer cell lines through the inhibition of different growth factors, enzymes and kinases including telomerase, histone deacetylase (HDAC), methionine aminopeptidase (MetAP), thymidylate synthase (TS), glycogen synthase kinase-3 (GSK), epidermal growth factor (EGF), vascular endothelial growth factor (VEGF), and focal adhesion kinase (FAK) [24,25,26]. Triazoles [27], tetrazoles [28], thiadiazoles [29], and pyrimidines [4] have also been reported to show anticancer activity.

Prompted by the aforementioned findings and in the continuation of our ongoing research in the field of design, synthesis, and biological evaluation of pyrazoline derivatives [30,31,32,33,34,35], herein we described the synthesis and evaluation of a new series of heteroaryl substituted pyrazolines as potential anticancer agents against AsPC-1 human pancreatic adenocarcinoma and two glioblastoma cell lines, U87 and U251 cell lines. Furthermore, tumor selectivity test on blood cells (PBMC and Jurkat cells) and the apoptotic, necrotic, and DNA-cleavage analysis against U251 cells were carried out using the most effective compound.

## 2. Results and Discussion

**Scheme 1:**
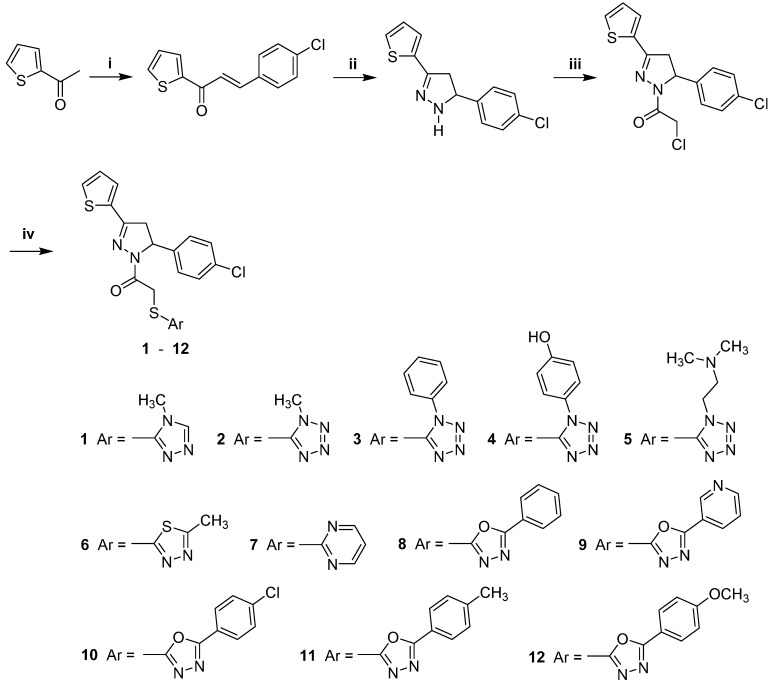
The synthesis of compounds **1**–**12**. *Reagents and conditions*: (i) 4-Chlorobenzaldehyde, 10% aqueous sodium hydroxide solution, ethanol, rt, 6–8 h; (ii) 80% hydrazine hydrate, ethanol, reflux, 5 h; (iii) ClCOCH_2_Cl, TEA, toluene, rt, 1 h; (iv) Ar-SH, acetone, rt, 8 h.

The synthesis of new pyrazoline derivatives (**1**–**12**) was carried out according to the steps shown in Scheme 1. In the initial step, 1-(2-thienyl)-3-(4-chlorophenyl)-2-propen-1-one was synthesized via the base-catalyzed Claisen-Schmidt condensation of 2-acetylthiophene with 4-chlorobenzaldehyde. The ring closure reaction of the chalcone with hydrazine hydrate afforded 5-(4-chlorophenyl)-3-(2-thienyl)-2-pyrazoline. 1-(Chloroacetyl)-3-(2-thienyl)-5-(4-chlorophenyl)-2-pyrazoline was obtained by the reaction of 5-(4-chlorophenyl)-3-(2-thienyl)-2-pyrazoline with chloroacetyl chloride in the presence of triethylamine. The reaction of 1-(chloroacetyl)-3-(2-thienyl)-5-(4-chlorophenyl)-2-pyrazoline with aryl thiols afforded 1-[(aryl)thioacetyl]-3-(2-thienyl)-5-(4-chlorophenyl)-2-pyrazolines (**1**–**12**). Thus, the synthetic procedure was shown to be versatile, applicable to the preparation of many derivatives.

The structures of the compounds were elucidated by IR, ^1^H-NMR, ^13^C-NMR, mass spectral data, and elemental analyses. In the IR spectra of compounds **1**–**12**, all derivatives had a strong, characteristic band in the region 1670–1641 cm^−1^ due to C=O stretching vibration. The asymmetric and symmetric stretching bands for aliphatic C–H group occurred at 2983–2920 cm^−1^. The aromatic C–H stretching vibrations gave rise to a band at 3134–3078 cm^−1^. The C=C, C=N and C–N stretching bands appeared in the region of 1548–1408 and 1398–1012 cm^−1^, respectively.

**Figure 1:**
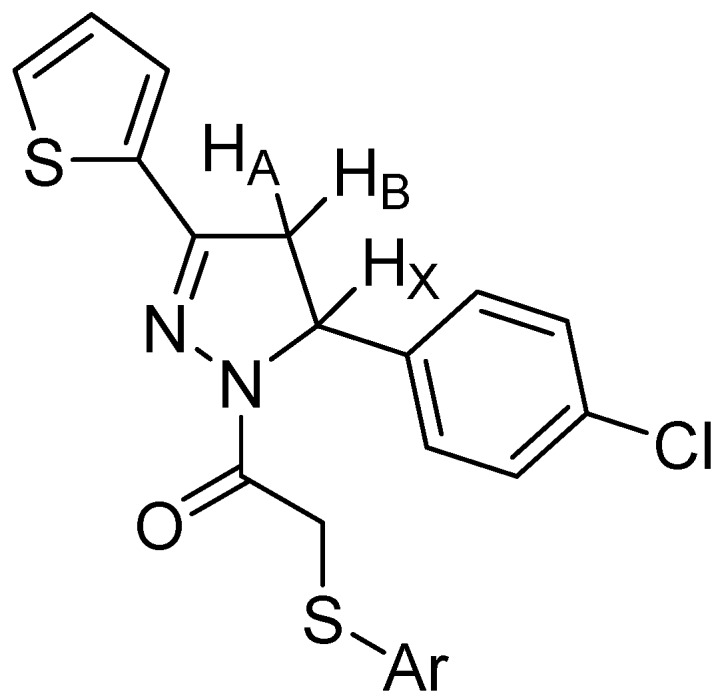
ABX system of the pyrazoline ring.

In the ^1^H-NMR spectra of the compounds, the CH_2_ protons of the pyrazoline ring resonated as a pair of doublets at δ 3.16–3.24 ppm (C_4_-H_A_), 3.89–3.94 ppm (C_4_-H_B_). The CH proton appeared as a doublet of doublets at δ 5.57–5.63 ppm (H_X_) due to the vicinal coupling with two magnetically non-equivalent protons of the methylene group at position four of the pyrazoline ring (*J*_AB_ = 17.60–18.00 Hz, *J*_AX_ = 4.00–4.80 Hz, *J*_BX_ = 11.20–12.00 Hz). The CH_2_ protons of the acetyl group at position 1 of the pyrazoline ring were observed at 4.32–4.83 ppm as a doublet (*J* = 15.20–16.40 Hz). This geminal coupling resulted from the steric structure of the compound. These geminal protons were observed as a doublet due to two different possible conformations since rigid protons occurred (Figure 1). All the other aromatic and aliphatic protons were observed at expected regions.

In the ^13^C-NMR spectra of the compounds, the signal due to the carbonyl carbon appeared at 163.92–166.37 ppm. The ^13^C-NMR chemical shift values of the carbon atoms at 43.34–43.42 ppm (C_4_), 60.04–60.24 ppm (C_5_) and 151.47–152.38 ppm (C_3_) corroborate 2-pyrazoline character deduced from the ^1^H-NMR data. The signal due to the S-CH_2_ carbon was observed in the region 34.30–39.72 ppm. The other aromatic and aliphatic carbons were observed at expected regions. The mass spectral data of the synthesized compounds were found in full agreement with the proposed structures. All compounds gave satisfactory elemental analysis.

**Figure 2:**
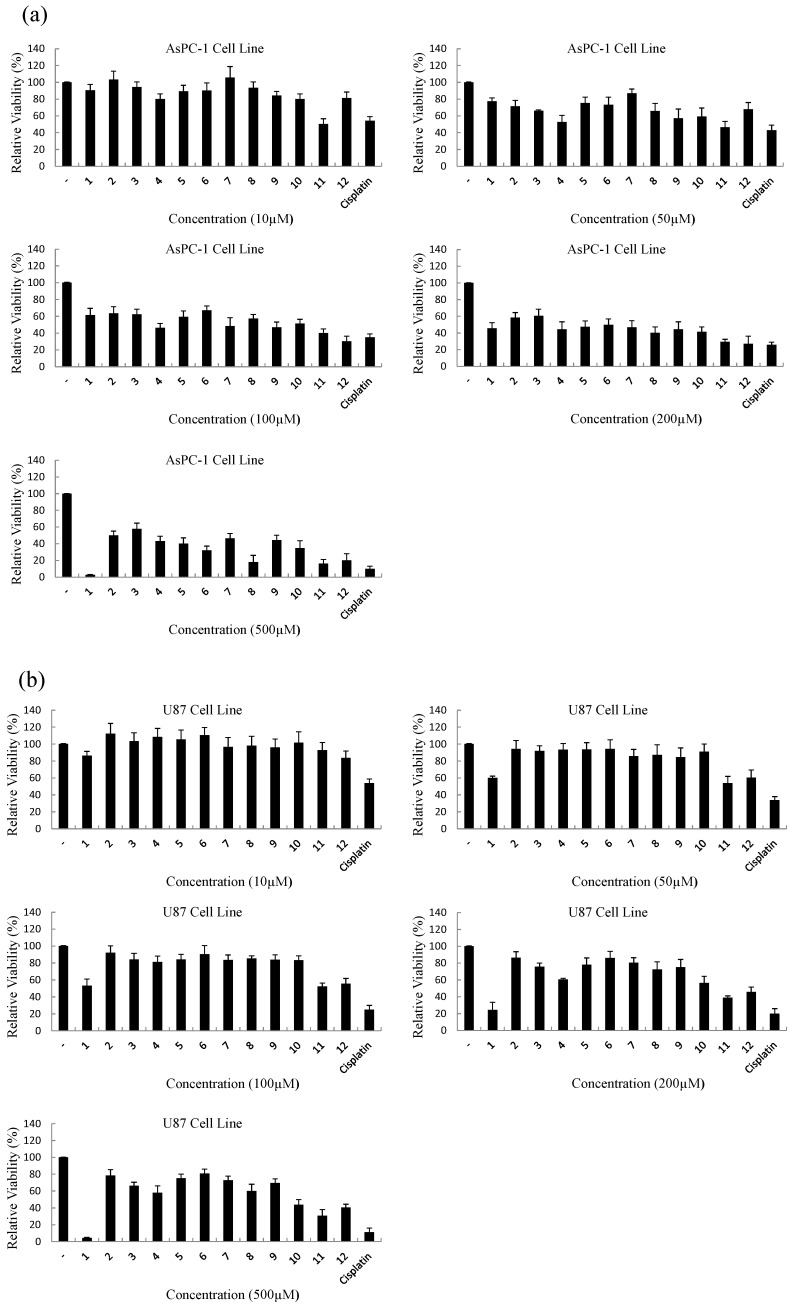

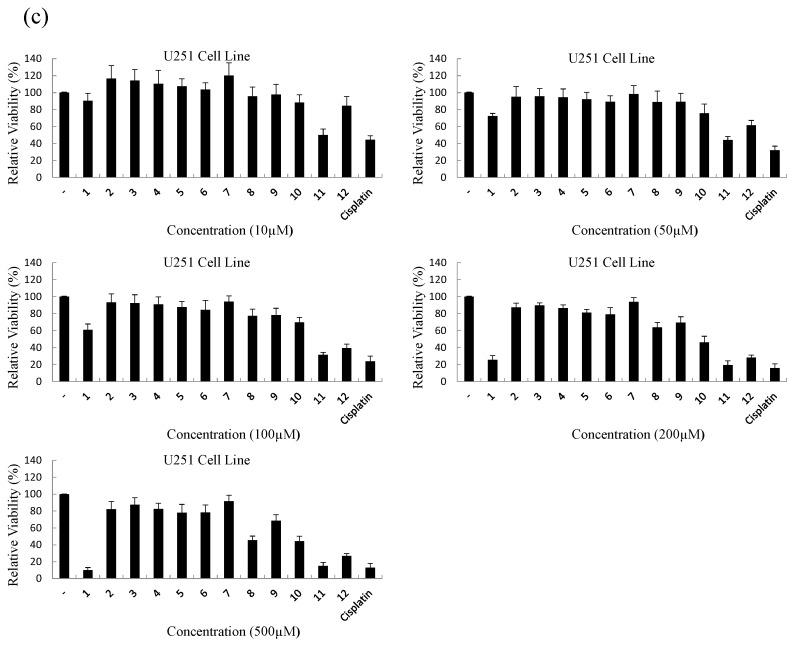
Anticancer effects of compounds **1**–**12** and cisplatin at varying concentrations (10 µM, 50 µM, 100 µM, 200 µM, and 500 µM) against AsPC-1 (**a**); U87 (**b**); and U251 (**c**) cells.

The anticancer effects of new pyrazoline derivatives (**1**–**12**) and cisplatin (positive control) in the range of 10–500 µM concentrations were tested. Since only limited choice of drugs are available for pancreatic cancer and glioma, AsPC-1 human pancreatic adenocarcinoma, U87 and U251 glioblastoma cell lines were used (Figure 2). Cisplatin was chosen as a control, considering its wide use in the treatment of several types of human cancer.

Compounds **1**–**12** were evaluated for their cytotoxic effects on these cell lines by MTT assay (Figure 2 and Table 1), to determine their anticancer potential and selectivity. The activity of the tested compounds was influenced considerably by the nature of the aryl group. Compounds **1**, **10**, **11**, and **12** were found to possess IC_50_ values lower than 500 μM against all three cell lines. Tetrazole-substituted compound **2** did not show activity, whereas triazole-substituted compound **1** was active. Generally, oxadiazole-substituted compounds **10**, **11**, and **12** exhibited good activity. The most effective cytotoxic agent against AsPC-1 and U251 cancer cell lines was found to be compound **11** with IC_50_ values of 16.8 µM, and 11.9 µM respectively, followed by compound **12** with respective IC_50_ values of 62.1 µM, and 70.1 µM. On the other hand, these compounds showed no significant cytotoxicity at the concentrations used for the U87 cell line.

**Table 1:** The cytotoxic effects of the compounds **1**–**12** on the AsPC-1, U87, and U251 cell lines.

Compound	IC_50_ (μM)		
	AsPC1 Cell Line	U87 Cell Line	U251 Cell Line
**1**	166.7 ± 10.6	112.2 ± 8.8	126.9 ± 15.6
**2**	>500	>500	>500
**3**	>500	>500	>500
**4**	65.0 ± 5.4	>500	>500
**5**	215.7 ± 29.7	>500	>500
**6**	199.3 ± 32.8	>500	>500
**7**	236.2 ± 36.4	>500	>500
**8**	139.3 ± 26.8	>500	393.1 ± 60.4
**9**	108.1 ± 30.1	>500	>500
**10**	110.5±20.4	250.6 ± 30.4	166.6 ± 25.1
**11**	16.8 ± 2.1	127.4 ± 12.8	11.9 ± 1.1
**12**	62.1 ± 7.8	159.2 ± 20.7	70.1 ± 8.8
**Cisplatin**	22.5 ± 2.0	14.8 ± 1.4	4.9 ± 1.0

**Figure 3:**
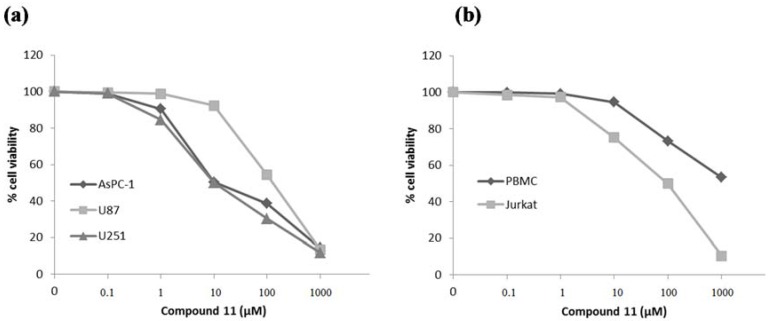
Effects of compound **11** on cell viability of AsPC-1, U87, U251 cell lines (**a**) and PBMC, Jurkat cell lines (**b**).

Among the tested compounds, compound **11** can be identified as the most promising anticancer agent. Thus, detail of concentration dependency of this compound (0.1 µM–1000 µM) was examined against AsPC-1, U87 and U251 cell lines (Figure 3a). Swelling ratio curves of AsPC-1 and U251 cell lines suggested biphasic mechanism of compound **11**. Moreover, the activity of compound **11** against peripheral blood mononuclear cells (PBMC) (IC_50_ = ~1000 μM) and Jurkat human leukemic T-cells (IC_50_ = 90 μM) indicated significant tumor selectivity in blood cells (more than 10 times) as shown in Figure 3b. This outcome pointed out the importance of 5-(4-methylphenyl)-1,3,4-oxadiazol-2-yl moiety for anticancer activity.

**Figure 4:**
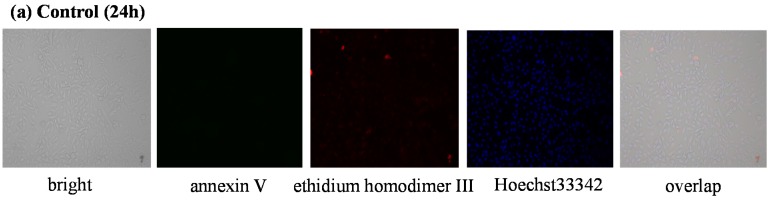

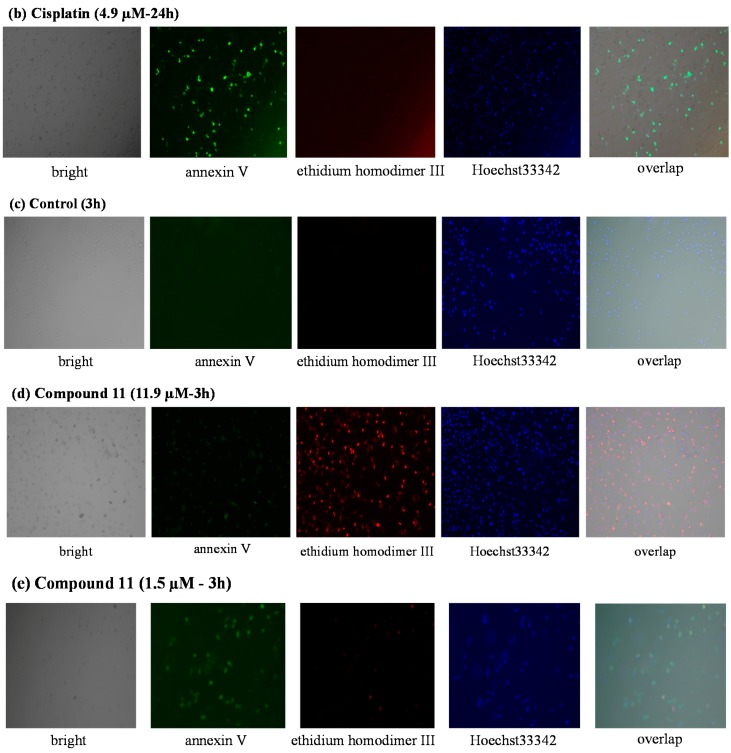
Cellular and nuclear morphological changes of U251 cells following exposure to IC_50_ concentrations of control (**a**); cisplatin (4.9 μM) (**b**); for 24 h and control (**c**); compound **11** (11.9 μM) (**d**); and (1.5 μM) (**e**) for 3 h.

According to the MTT assay results, the most active anticancer compound **11** was chosen for the evaluation of apoptosis and necrosis in U251 cells, which was carried out with annexin V/ethidium homodimer III staining method. U251 cell lines were incubated with compound **11** or cisplatin at the IC_50_ concentrations. U251 cells were stained and observed by fluorescence microscope (Figure 4). If the cells are colored green with annexin V, and not stained red with ethidium homodimer III, the cells are judged to be apoptotic. On the other hand, the completely opposite results indicate necrosis. The apoptotic and necrotic effects of compound **11** were compared with cisplatin. The results indicate that cisplatin has only apoptotic effects at 24 h (Figure 4b). In contrast, U251 cell lines treated with compound **11** at 24 h showed late stage apoptotic or necrotic effect (almost all cells were colored yellow, data not shown). Therefore, the apoptotic/necrotic effects of compound **11** were tested against U251 cell line in earlier time (3h) at IC_50_ (11.9 µM) and low (1.5 µM) concentrations. While compound **11** mainly induced necrosis at high concentration (Figure 4d), it induced apoptosis at low concentration (Figure 4e).

**Figure 5:**
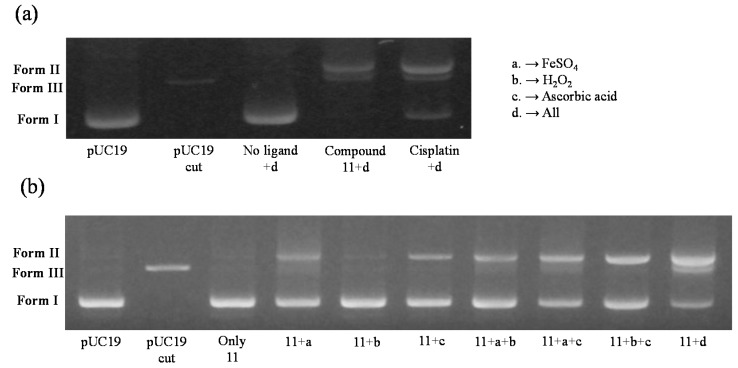
The DNA cleaving capability of the Fe(II) complexes of compound **11** and cisplatin in the presence of the iron (II) complex (**a**) and control experiments using compound **11** in the presence and absence of FeSO_4_, H_2_O_2_, and ascorbic acid (**b**). It was studied by the relaxation of the supercoiled pUC19 DNA and analyzed by agarose-gel electrophoresis.

The DNA cleavage activities of compound **11** and cisplatin at the IC_50_ concentrations in water and Tris/boric acid buffer in the presence and absent of the iron complex, H_2_O_2_ and ascorbic acid as an activator were studied using supercoiled pUC19 DNA (Figure 5). The reaction mixture was incubated at 37 °C for 1.5 h and then agarose gel electrophoresis was performed at 100 V for 40 min. DNA was visualized by photographing the fluorescence of intercalated ethidium bromide under a UV illuminator. Control experiments using compound **11** with and without FeSO_4_, H_2_O_2_, and ascorbic acid showed that the DNA cleavage efficiency was clearly enhanced in the case of iron (II) complex system (Figure 5b). Thus, it is considered that DNA-cleavage was caused by oxygen activation. The DNA-cleaving efficiency of compound **11** was much greater than cisplatin. Compound **11** disintegrated pUC 19 DNA and these results suggest the relationship between the DNA cleavage and the cell death.

## 3. Experimental Section

### 3.1. Chemistry

All reagents were purchased from commercial suppliers and were used without further purification. Melting points were determined on an Electrothermal 9100 melting point apparatus (Weiss-Gallenkamp, Loughborough, UK) and were uncorrected. IR spectra were recorded on a Shimadzu 8400 FT-IR spectrophotometer (Shimadzu, Tokyo, Japan). ^1^H-NMR and ^13^C-NMR spectra were recorded on a Varian Mercury-400 FT-NMR spectrometer (Agilent, Palo Alto, CA, USA). Mass spectra were recorded on an Agilent LC-MSD-Trap-SL Mass spectrometer (Agilent Technologies). Elemental analyses were performed on a Perkin Elmer EAL 240 elemental analyzer (Perkin-Elmer, Norwalk, CT, USA). Thin Layer Chromatography (TLC) was performed on TLC silica gel 60 F_254_ aluminium sheets (Merck, Darmstadt, Germany) using petroleum ether:ethyl acetate (3:1 *v*/*v*) as an eluent.

#### General Procedures for the Synthesis of Compounds

1-(2-Thienyl)-3-(4-chlorophenyl)-2-propen-1-one

A mixture of 2-acetylthiophene (0.04 mol), 4-chlorobenzaldehyde (0.04 mol) and 10% aqueous sodium hydroxide solution (10 mL) in ethanol (30 mL) was stirred at room temperature for 6–8 h. The progress of the reaction was checked by TLC. Upon completion, the reaction mixture was poured into crushed ice. The precipitated solid was filtered, washed with water, and dried. The product was crystallized from ethanol [36].

5-(4-Chlorophenyl)-3-(2-thienyl)-2-pyrazoline

A mixture of 1-(2-thienyl)-3-(4-chlorophenyl)-2-propen-1-one (0.01 mol) and hydrazine hydrate (80%) (0.02 mol) in ethanol (30 mL) was refluxed for 5 h. The reaction mixture was cooled and kept at 0 °C overnight. The resulting solid was filtered and dried. The product was crystallized from ethanol [36].

1-(Chloroacetyl)-3-(2-thienyl)-5-(4-chlorophenyl)-2-pyrazoline

5-(4-Chlorophenyl)-3-(2-thienyl)-2-pyrazoline (0.01 mol) and triethylamine (0.01 mol) were dissolved in toluene (30 mL). The reaction mixture was cooled in an ice bath, and chloroacetyl chloride (0.01 mol) was added dropwise with constant stirring. The mixture thus obtained was further agitated for 1 h at room temperature. The solvent was evaporated to dryness under reduced pressure. The residue was washed with water, and the product was recrystallized from ethanol [36].

*1-[(Aryl)thioacetyl]-3-(2-thienyl)-5-(4-chlorophenyl)-2-pyrazolines* (**1**–**12**)

A mixture of 1-(chloroacetyl)-3-(2-thienyl)-5-(4-chlorophenyl)-2-pyrazoline (0.005 mol) and aryl thiol (0.005 mol) was treated in acetone (30 mL) at room temperature for 8 h. The solvent was evaporated, the residue was washed with water and recrystallized from ethanol [37].

*1-[(4-Methyl-4H-1,2,4-triazol-3-yl)thioacetyl]-3-(2-thienyl)-5-(4-chlorophenyl)-2-pyrazoline* (**1**). Yield: 83%; M.p. 165.1 °C. IR (KBr) ν_max_ (cm^−1^): 3124 (aromatic C-H), 2922 (aliphatic C-H asymmetric), 1666 (C=O), 1490, 1458, 1417 (C=N and C=C), 1375, 1203, 1085, 1026 (C-N), 952, 817 (C-H out of plane deformation). ^1^H-NMR (400 MHz, δ ppm, DMSO-*d*_6_): 3.16 (1H, dd, *J*_AX_ = 4.40 Hz, *J*_AB_ = 17.60 Hz, pyrazoline C_4_-H_A_), 3.58 (3H, CH_3_), 3.89 (1H, dd, *J*_BX_ = 11.60 Hz, *J*_BA_ = 18.00 Hz, pyrazoline C_4_-H_B_), 4.33 (1H, d, *J* = 15.20 Hz, geminal proton, CO-C*H*), 4.46 (1H, d, *J* = 15.60 Hz, geminal proton, CO-C*H*), 5.57 (1H, dd, *J*_AX_ = 4.80 Hz, *J*_BX_ = 11.60 Hz, pyrazoline C_5_-H_X_), 7.16 (1H, m), 7.26 (2H, d, *J* = 8.80 Hz), 7.38 (2H, d, *J* = 8.80 Hz), 7.48 (1H, dd, *J* = 1.20 Hz, 4.00 Hz, thiophene C_3_-H), 7.78 (1H, dd, *J* = 1.20 Hz, 5.20 Hz, thiophene C_5_-H), 8.52 (1H, s, triazole). ^13^C-NMR (100 MHz, DMSO-*d*_6_) δ (ppm): 31.49 (CH_3_, triazole), 39.72 (CH_2_, S-CH_2_), 43.36 (CH_2_, pyrazoline C_4_), 60.04 (CH, pyrazoline C_5_), 128.27 (2CH, aromatic), 128.81 (CH, aromatic), 129.28 (2CH, aromatic), 130.83 (C, aromatic), 131.73 (CH, aromatic), 132.61 (CH, aromatic), 134.23 (C, aromatic), 141.05 (C, aromatic), 146.94 (CH, aromatic), 149.16 (C, aromatic), 151.95 (C, pyrazoline C_3_), 164.96 (C, C=O). Anal. Calcd. for C_18_H_16_ClN_5_OS_2_: C, 51.73; H, 3.86; N, 16.76. Found: C, 51.66; H, 3.89; N, 16.81. MS (ESI) (*m*/*z*): 418 [M + H]^+^.

*1-[(1-Methyl-1H-tetrazol-5-yl)thioacetyl]-3-(2-thienyl)-5-(4-chlorophenyl)-2-pyrazoline* (**2**). Yield: 86%; M.p. 108.2 °C. IR (KBr) ν_max_ (cm^−1^): 3107 (aromatic C-H), 2941 (aliphatic C-H asymmetric), 1658 (C=O), 1463, 1417 (C=N and C=C), 1396, 1278, 1219, 1089, 1026 (C-N), 968, 837, 819 (C-H out of plane deformation). ^1^H-NMR (400 MHz, δ ppm, DMSO-*d*_6_): 3.22 (1H, dd, *J*_AX_ = 4.80 Hz, *J*_AB_ = 18.40 Hz, pyrazoline C_4_-H_A_), 3.91 (1H, dd, *J*_BX_ = 11.60 Hz, *J*_BA_ = 18.00 Hz, pyrazoline C_4_-H_B_), 3.95 (3H, CH_3_), 4.57 (1H, d, *J* = 15.60 Hz, geminal proton, CO-C*H*), 4.71 (1H, d, *J* = 16.00 Hz, geminal proton, CO-C*H*), 5.59 (1H, dd, *J*_AX_ = 4.80 Hz, *J*_BX_ = 12.00 Hz, pyrazoline C_5_-H_X_), 7.17 (1H, m), 7.26 (2H, d, *J* = 8.40 Hz), 7.38 (2H, d, *J* = 8.80 Hz), 7.50 (1H, dd, *J* = 1.20 Hz, 3.60 Hz, thiophene C_3_-H), 7.79 (1H, dd, *J* = 0.80 Hz, 5.20 Hz, thiophene C_5_-H). ^13^C-NMR (100 MHz, DMSO-*d*_6_) δ (ppm): 34.32 (CH_3_, tetrazole), 37.15 (CH_2_, S-CH_2_), 43.40 (CH_2_, pyrazoline C_4_), 60.16 (CH, pyrazoline C_5_), 128.26 (2CH, aromatic), 128.84 (CH, aromatic), 129.29 (2CH, aromatic), 130.96 (C, aromatic), 131.90 (CH, aromatic), 132.66 (CH, aromatic), 134.14 (C, aromatic), 140.96 (C, aromatic), 152.29 (C, pyrazoline C_3_), 153.97 (C, aromatic), 164.19 (C, C=O). Anal. Calcd. for C_17_H_15_ClN_6_OS_2_: C, 48.74; H, 3.61; N, 20.06. Found: C, 48.65; H, 3.68; N, 19.95. MS (ESI) (*m*/*z*): 419 [M + H]^+^, 420 [M + H]^++^.

*1-[(1-Phenyl-1H-tetrazol-5-yl)thioacetyl]-3-(2-thienyl)-5-(4-chlorophenyl)-2-pyrazoline* (**3**). Yield: 84%; M.p. 183.3 °C. IR (KBr) ν_max_ (cm^−1^): 3107 (aromatic C-H), 2933 (aliphatic C-H asymmetric), 1656 (C=O), 1498, 1462, 1423 (C=N and C=C), 1388, 1220, 1089, 1012 (C-N), 831 (C-H out of plane deformation). ^1^H-NMR (400 MHz, δ ppm, DMSO-*d*_6_): 3.23 (1H, dd, *J*_AX_ = 4.40 Hz, *J*_AB_ = 18.00 Hz, pyrazoline C_4_-H_A_), 3.94 (1H, dd, *J*_BX_ = 11.60 Hz, *J*_BA_ = 18.00 Hz, pyrazoline C_4_-H_B_), 4.69 (1H, d, *J* = 16.00 Hz, geminal proton, CO-C*H*), 4.83 (1H, d, *J* = 16.40 Hz, geminal proton, CO-C*H*), 5.60 (1H, dd, *J*_AX_ = 4.00 Hz, *J*_BX_ = 11.20 Hz, pyrazoline C_5_-H_X_), 7.18 (1H, m), 7.27 (2H, d, *J* = 8.40 Hz), 7.38 (2H, d, *J* = 8.80 Hz), 7.51 (1H, dd, *J*=1.20 Hz, 3.60 Hz, thiophene C_3_-H), 7.64–7.68 (5H, m), 7.80 (1H, dd, *J* = 1.20 Hz, 4.80 Hz, thiophene C_5_-H). ^13^C-NMR (100 MHz, DMSO-*d*_6_) δ (ppm): 37.35 (CH_2_, S-CH_2_), 43.41 (CH_2_, pyrazoline C_4_), 60.21 (CH, pyrazoline C_5_), 125.10 (2CH, aromatic), 128.27 (2CH, aromatic), 128.87 (CH, aromatic), 129.29 (2CH, aromatic), 130.75 (2CH, aromatic), 131.01 (CH, aromatic), 131.34 (CH, aromatic), 131.98 (CH, aromatic), 132.65 (C, aromatic), 133.70 (C, aromatic), 134.13 (C, aromatic), 140.96 (C, aromatic), 152.38 (C, pyrazoline C_3_), 154.65 (C, aromatic), 163.92 (C, C=O). Anal. Calcd. for C_22_H_17_ClN_6_OS_2_: C, 54.94; H, 3.56; N, 17.47. Found: C, 54.99; H, 3.58; N, 17.51. MS (ESI) (*m*/*z*): 481 [M + H]^+^.

*1-[(1-(4-Hydroxyphenyl)-1H-tetrazol-5-yl)thioacetyl]-3-(2-thienyl)-5-(4-chlorophenyl)-2-pyrazoline* (**4**). Yield: 71%; M.p. 145.7 °C. IR (KBr) ν_max_ (cm^−1^): 3601 (O-H), 3107 (aromatic C-H), 2932 (aliphatic C-H asymmetric), 1641 (C=O), 1516, 1454, 1415 (C=N and C=C), 1278, 1228, 1087 (C-N), 837, 823 (C-H out of plane deformation). ^1^H-NMR (400 MHz, δ ppm, DMSO-*d*_6_): 3.22 (1H, dd, *J*_AX_ = 4.80 Hz, *J*_AB_ = 18.00 Hz, pyrazoline C_4_-H_A_), 3.93 (1H, dd, *J*_BX_ = 11.60 Hz, *J_BA_* = 18.00 Hz, pyrazoline C_4_-H_B_), 4.65 (1H, d, *J* = 16.00 Hz, geminal proton, CO-C*H*), 4.77 (1H, d, *J* = 16.00 Hz, geminal proton, CO-C*H*), 5.60 (1H, dd, *J*_AX_ = 4.40 Hz, *J*_BX_ = 11.60 Hz, pyrazoline C_5_-H_X_), 6.87 (2H, d, *J* = 8.80 Hz), 7.17 (1H, m), 7.26–7.33 (4H, m), 7.37 (2H, d, *J* = 8.40 Hz), 7.51 (1H, d, *J* = 3.60 Hz, thiophene C_3_-H), 7.79 (1H, d, *J* = 5.20 Hz, thiophene C_5_-H), 9.35 (1H, s). ^13^C-NMR (100 MHz, DMSO-*d*_6_) δ (ppm): 37.04 (CH_2_, S-CH_2_), 43.40 (CH_2_, pyrazoline C_4_), 60.20 (CH, pyrazoline C_5_), 117.35 (2CH, aromatic), 122.57 (C, aromatic), 126.65 (2CH, aromatic), 128.27 (2CH, aromatic), 128.85 (CH, aromatic), 129.29 (2CH, aromatic), 130.96 (CH, aromatic), 131.90 (CH, aromatic), 132.64 (CH, aromatic), 134.16 (C, aromatic), 140.98 (C, aromatic), 152.28 (C, pyrazoline C_3_), 154.65 (C, aromatic), 163.04 (C, aromatic), 164.05 (C, C=O). Anal. Calcd. for C_22_H_17_ClN_6_O_2_S_2_: C, 53.17; H, 3.45; N, 16.91. Found: C, 53.21; H, 3.54; N, 16.96. MS (ESI) (*m*/*z*): 497 [M + H]^+^, 498 [M + H]^++^.

*1-[(1-(2-(Dimethylamino)ethyl)-1H-tetrazol-5-yl)thioacetyl]-3-(2-thienyl)-5-(4-chlorophenyl)-2-pyrazoline* (**5**). Yield: 76%; M.p. 112.1 °C. IR (KBr) ν_max_ (cm^−1^): 3091 (aromatic C-H), 2943, 2827 (aliphatic C-H asymmetric), 1654 (C=O), 1452, 1413 (C=N and C=C), 1394, 1220, 1093, 1029 (C-N), 831, 817 (C-H out of plane deformation). ^1^H-NMR (400 MHz, δ ppm, DMSO-*d*_6_): 2.13 (6H, s), 2.64 (2H, t, *J* = 6.40 Hz, 12.00 Hz), 3.22 (1H, dd, *J*_AX_ = 4.80 Hz, *J*_AB_ = 18.00 Hz, pyrazoline C_4_-H_A_), 3.92 (1H, dd, *J*_BX_ = 11.60 Hz, *J*_BA_ = 17.60 Hz, pyrazoline C_4_-H_B_), 4.38 (2H, t, *J* = 6.00 Hz, 12.00 Hz), 4.57 (1H, d, *J* = 15.60 Hz, geminal proton, CO-C*H*), 4.71 (1H, d, *J* = 16.00 Hz, geminal proton, CO-C*H*), 5.59 (1H, dd, *J*_AX_ = 4.80 Hz, *J*_BX_ = 12.00 Hz, pyrazoline C_5_-H_X_), 7.17 (1H, m), 7.26 (2H, d, *J* = 8.80 Hz), 7.38 (2H, d, *J* = 8.80 Hz), 7.49 (1H, dd, *J* = 0.80, 3.60 Hz, thiophene C_3_-H), 7.79 (1H, dd, *J* = 5.20, 0.80 Hz, thiophene C_5_-H). ^13^C-NMR (100 MHz, DMSO-*d*_6_) δ (ppm): 37.46 (CH_2_, S-CH_2_), 43.38 (CH_2_, pyrazoline C_4_), 45.65 (2CH_3_, H_3_C-N-CH_3_), 45.99 (CH_2_, CH_2_-tetrazole), 57.84 (CH_2_, CH_2_-N(CH_3_)_2_), 60.15 (CH, pyrazoline C_5_), 128.25 (2CH, aromatic), 128.84 (CH, aromatic), 129.29 (2CH, aromatic), 130.97 (CH, aromatic), 131.91 (CH, aromatic), 132.64 (C, aromatic), 134.15 (C, aromatic), 140.99 (C, aromatic), 152.25 (C, pyrazoline C_3_), 154.19 (C, aromatic), 164.23 (C, C=O). Anal. Calcd. for C_20_H_22_ClN_7_OS_2_: C, 50.46; H, 4.66; N, 20.60. Found: C, 50.42; H, 4.69; N, 20.57. MS (ESI) (*m*/*z*): 476 [M], 477 [M + H]^+^, 478 [M + H]^++^.

*1-[(5-Methyl-1,3,4-thiadiazol-2-yl)thioacetyl]-3-(2-thienyl)-5-(4-chlorophenyl)-2-pyrazoline* (**6**). Yield: 82%; M.p. 140.9 °C. IR (KBr) ν_max_ (cm^−1^): 3103 (aromatic C-H), 2920 (aliphatic C-H asymmetric), 1645 (C=O), 1456, 1417 (C=N and C=C), 1398, 1325, 1219, 1072, 1029 (C-N), 840, 819 (C-H out of plane deformation). ^1^H-NMR (400 MHz, δ ppm, DMSO-*d*_6_): 2.66 (3H, s), 3.22 (1H, dd, *J*_AX_ = 4.80 Hz, *J*_AB_ = 18.00 Hz, pyrazoline C_4_-H_A_), 3.92 (1H, dd, *J*_BX_ = 11.60 Hz, *J*_BA_ = 17.60 Hz, pyrazoline C_4_-H_B_), 4.54 (1H, d, *J* = 16.00 Hz, geminal proton, CO-C*H*), 4.67 (1H, d, *J* = 16.00 Hz, geminal proton, CO-C*H*), 5.60 (1H, dd, *J*_AX_ = 4.40 Hz, *J*_BX_ = 11.60 Hz, pyrazoline C_5_-H_X_), 7.17 (1H, m), 7.27 (2H, d, *J* = 8.40 Hz), 7.38 (2H, d, *J* = 8.80 Hz), 7.50 (1H, dd, *J* = 0.80 Hz, 3.60 Hz, thiophene C_3_-H), 7.79 (1H, dd, *J* = 1.20 Hz, 5.20 Hz, thiophene C_5_-H). ^13^C-NMR (100 MHz, DMSO-*d*_6_) δ (ppm): 15.85 (CH_3_, thiadiazole), 37.33 (CH_2_, S-CH_2_), 43.36 (CH_2_, pyrazoline C_4_), 60.13 (CH, pyrazoline C_5_), 128.29 (2CH, aromatic), 128.83 (CH, aromatic), 129.29 (2CH, aromatic), 130.93 (CH, aromatic), 131.83 (CH, aromatic), 132.63 (C, aromatic), 134.20 (C, aromatic), 141.05 (C, aromatic), 152.08 (C, pyrazoline C_3_), 164.40 (C, aromatic), 164.98 (C, aromatic), 166.37 (C, C=O). Anal. Calcd. for C_18_H_15_ClN_4_OS_3_: C, 49.70; H, 3.48; N, 12.88. Found: C, 49.72; H, 3.49; N, 12.83. MS (ESI) (*m*/*z*): 434 [M], 435 [M + H]^+^, 436 [M + H]^++^, 437 [M + H]^+++^.

*1-[(Pyrimidin-2-yl)thioacetyl]-3-(2-thienyl)-5-(4-chlorophenyl)-2-pyrazoline* (**7**). Yield: 78%; M.p. 126.6 °C. IR (KBr) ν_max_ (cm^−1^): 3109 (aromatic C-H), 2929 (aliphatic C-H asymmetric), 1653 (C=O), 1548, 1448, 1409 (C=N and C=C), 1379, 1201, 1176, 1089, 1016 (C-N), 821 (C-H out of plane deformation). ^1^H-NMR (400 MHz, δ ppm, DMSO-*d*_6_): 3.19 (1H, dd, *J*_AX_ = 4.40 Hz, *J*_AB_ = 18.00 Hz, pyrazoline C_4_-H_A_), 3.92 (1H, dd, *J*_BX_ = 12.00 Hz, *J*_BA_ = 17.60 Hz, pyrazoline C_4_-H_B_), 4.32 (1H, d, *J* = 16.40 Hz, geminal proton, CO-C*H*), 4.60 (1H, d, *J* = 16.00 Hz, geminal proton, CO-C*H*), 5.61 (1H, dd, *J*_AX_ = 4.80 Hz, *J*_BX_ = 11.60 Hz, pyrazoline C_5_-H_X_), 7.15–7.18 (1H, m), 7.22 (1H, t, *J* = 4.80 Hz, 5.20 Hz, pyrimidine), 7.28 (2H, d, *J* = 8.00 Hz), 7.40 (2H, d, *J* = 8.00 Hz), 7.47–7.49 (1H, m, thiophene C_3_-H), 7.77 (1H, dd, *J* = 0.80 Hz, 4.40 Hz, thiophene C_5_-H), 8.63 (2H, d, *J* = 4.80 Hz, pyrimidine). ^13^C-NMR (100 MHz, DMSO-*d*_6_) δ (ppm): 34.30 (CH_2_, S-CH_2_), 43.34 (CH_2_, pyrazoline C_4_), 60.09 (CH, pyrazoline C_5_), 118.01 (CH, aromatic), 128.13 (2CH, aromatic), 128.81 (CH, aromatic), 129.27 (2CH, aromatic), 130.68 (CH, aromatic), 131.58 (CH, aromatic), 132.54 (C, aromatic), 134.43 (C, aromatic), 141.33 (C, aromatic), 151.47 (C, pyrazoline C_3_), 158.42 (2CH, aromatic), 165.28 (C, C=O), 170.99 (C, aromatic). Anal. Calcd. for C_19_H_15_ClN_4_OS_2_: C, 55.00; H, 3.64; N, 13.50. Found: C, 55.11; H, 3.51; N, 13.43. MS (ESI) (*m*/*z*): 414 [M], 415 [M + H]^+^, 416 [M + H]^++^.

*1-[(5-Phenyl-1,3,4-oxadiazol-2-yl)thioacetyl]-3-(2-thienyl)-5-(4-chlorophenyl)-2-pyrazoline* (**8**). Yield: 74%; M.p. 162.3 °C. IR (KBr) ν_max_ (cm^−1^): 3107 (aromatic C-H), 2983 (aliphatic C-H asymmetric), 1670 (C=O), 1465, 1450, 1408 (C=N and C=C), 1238, 1197, 1068, 1012 (C-N), 827 (C-H out of plane deformation). ^1^H-NMR (400 MHz, δ ppm, DMSO-*d*_6_): 3.23 (1H, dd, *J*_AX_ = 4.40 Hz, *J*_AB_ = 18.00 Hz, pyrazoline C_4_-H_A_), 3.93 (1H, dd, *J*_BX_ = 11.60 Hz, *J*_BA_ = 18.00 Hz, pyrazoline C_4_-H_B_), 4.63 (1H, d, *J* = 16.00 Hz, geminal proton, CO-C*H*), 4.77 (1H, d, *J* = 16.00 Hz, geminal proton, CO-C*H*), 5.62 (1H, dd, *J*_AX_ = 4.80 Hz, *J*_BX_ = 11.60 Hz, pyrazoline C_5_-H_X_), 7.16–7.19 (1H, m), 7.26 (2H, d, *J* = 8.40 Hz), 7.34 (2H, d, *J* = 8.80 Hz), 7.51–7.64 (4H, m), 7.78–7.80 (1H, m, thiophene C_5_-H), 7.92–7.95 (2H, m). ^13^C-NMR (100 MHz, DMSO-*d_6_*) δ (ppm): 36.11 (CH_2_, S-CH_2_), 43.40 (CH_2_, pyrazoline C_4_), 60.19 (CH, pyrazoline C_5_), 123.66 (C, aromatic), 127.03 (2CH, aromatic), 128.24 (2CH, aromatic), 128.85 (CH, aromatic), 129.29 (2CH, aromatic), 130.07 (2CH, aromatic), 131.00 (CH, aromatic), 131.95 (CH, aromatic), 132.66 (C, aromatic), 132.69 (CH, aromatic), 134.12 (C, aromatic), 140.99 (C, aromatic), 152.35 (C, pyrazoline C_3_), 164.02 (C, aromatic), 164.13 (C, aromatic), 165.77 (C, C=O). Anal. Calcd. for C_23_H_17_ClN_4_O_2_S_2_: C, 57.43; H, 3.56; N, 11.65. Found: C, 57.47; H, 3.55; N, 11.61. MS (ESI) (*m*/*z*): 481 [M + H]^+^.

*1-[(5-(Pyridin-3-yl)-1,3,4-oxadiazol-2-yl)thioacetyl]-3-(2-thienyl)-5-(4-chlorophenyl)-2-pyrazoline* (**9**). Yield: 70%; M.p. 156.9 °C. IR (KBr) ν_max_ (cm^−1^): 3080 (aromatic C-H), 2981 (aliphatic C-H asymmetric), 1668 (C=O), 1456, 1417 (C=N and C=C), 1199, 1076, 1016 (C-N), 833 (C-H out of plane deformation). ^1^H-NMR (400 MHz, δ ppm, DMSO-*d*_6_): 3.24 (1H, dd, *J*_AX_ = 4.80 Hz, *J*_AB_ = 18.00 Hz, pyrazoline C_4_-H_A_), 3.94 (1H, dd, *J*_BX_ = 11.60 Hz, *J*_BA_ = 18.40 Hz, pyrazoline C_4_-H_B_), 4.65 (1H, d, *J* = 16.00 Hz, geminal proton, CO-C*H*), 4.79 (1H, d, *J* = 16.00 Hz, geminal proton, CO-C*H*), 5.63 (1H, dd, *J*_AX_ = 4.80 Hz, *J*_BX_ = 12.00 Hz, pyrazoline C_5_-H_X_), 7.16–7.18 (1H, m), 7.28 (2H, d, *J* = 8.40 Hz), 7.35 (2H, d, *J* = 8.40 Hz), 7.50 (1H, dd, *J* = 1.20 Hz, 4.00 Hz, thiophene C_3_-H), 7.58–7.62 (1H, m, pyridine C_5_-H), 7.78 (1H, dd, *J* = 0.80 Hz, 4.80 Hz, thiophene C_5_-H), 8.29-8.32 (1H, m, pyridine), 8.79 (1H, dd, *J* = 1.60 Hz, 4.80 Hz, pyridine), 9.12 (1H, d, *J* = 1.60 Hz, pyridine). ^13^C-NMR (100 MHz, DMSO-*d*_6_) δ (ppm): 36.28 (CH_2_, S-CH_2_), 43.42 (CH_2_, pyrazoline C_4_), 60.23 (CH, pyrazoline C_5_), 120.31 (CH, aromatic), 124.98 (C, aromatic), 128.27 (2CH, aromatic), 128.85 (CH, aromatic), 129.31 (2CH, aromatic), 130.99 (CH, aromatic), 131.93 (CH, aromatic), 132.69 (CH, aromatic), 134.12 (C, aromatic), 134.64 (C, aromatic), 140.99 (C, aromatic), 147.74 (CH, aromatic), 152.37 (C, pyrazoline C_3_), 153.16 (CH, aromatic), 164.04 (C, aromatic), 164.08 (C, aromatic), 164.76 (C, C=O). Anal. Calcd. for C_22_H_16_ClN_5_O_2_S_2_: C, 54.82; H, 3.35; N, 14.53. Found: C, 54.83; H, 3.33; N, 14.60. MS (ESI) (*m*/*z*): 481 [M], 482 [M + H]^+^, 483 [M + H]^++^.

*1-[(5-(4-Chlorophenyl)-1,3,4-oxadiazol-2-yl)thioacetyl]-3-(2-thienyl)-5-(4-chlorophenyl)-2-pyrazoline* (**10**). Yield: 77%; M.p. 176.5 °C. IR (KBr) ν_max_ (cm^−1^): 3099 (aromatic C-H), 2981 (aliphatic C-H asymmetric), 1643 (C=O), 1463, 1417 (C=N and C=C), 1392, 1188, 1174, 1082, 1008 (C-N), 952, 840, 819 (C-H out of plane deformation). ^1^H-NMR (400 MHz, δ ppm, DMSO-*d*_6_): 3.23 (1H, dd, *J*_AX_ = 4.80 Hz, *J*_AB_ = 18.40 Hz, pyrazoline C_4_-H_A_), 3.93 (1H, dd, *J*_BX_ = 11.60 Hz, *J*_BA_ = 18.00 Hz, pyrazoline C_4_-H_B_), 4.62 (1H, d, *J* = 16.00 Hz, geminal proton, CO-C*H*), 4.75 (1H, d, *J* = 16.00 Hz, geminal proton, CO-C*H*), 5.62 (1H, dd, *J*_AX_ = 4.80 Hz, *J*_BX_ = 11.20 Hz, pyrazoline C_5_-H_X_), 7.17 (1H, dd, *J* = 3.60 Hz, 5.20 Hz thiophene C_4_-H), 7.27 (2H, d, *J* = 8.40 Hz), 7.35 (2H, d, *J* = 8.40 Hz), 7.50 (1H, dd, *J* = 0.80 Hz, 3.60 Hz, thiophene C_3_-H), 7.62 (2H, d, *J* = 8.80 Hz), 7.77 (1H, dd, *J* = 0.80 Hz, 5.20 Hz, thiophene C_5_-H), 7.93 (2H, d, *J* = 8.40 Hz). ^13^C-NMR (100 MHz, DMSO-*d*_6_) δ (ppm): 36.16 (CH_2_, S-CH_2_), 43.41 (CH_2_, pyrazoline C_4_), 60.23 (CH, pyrazoline C_5_), 122.55 (C, aromatic), 128.25 (2CH, aromatic), 128.81 (3CH, aromatic), 129.29 (2CH, aromatic), 130.21 (2CH, aromatic), 130.93 (CH, aromatic), 131.82 (CH, aromatic), 132.71 (C, aromatic), 134.15 (C, aromatic), 137.42 (C, aromatic), 140.96 (C, aromatic), 152.32 (C, pyrazoline C_3_), 164.12 (C, aromatic), 164.30 (C, aromatic), 165.05 (C, C=O). Anal. Calcd. for C_23_H_16_Cl_2_N_4_O_2_S_2_: C, 53.59; H, 3.13; N, 10.87. Found: C, 53.48; H, 3.17; N, 10.75. MS (ESI) (*m*/*z*): 515 [M], 516 [M + H]^+^, 517 [M + H]^++^.

*1-[(5-(4-Methylphenyl)-1,3,4-oxadiazol-2-yl)thioacetyl]-3-(2-thienyl)-5-(4-chlorophenyl)-2-pyrazoline* (**11**). Yield: 79%; M.p. 194.0 °C. IR (KBr) ν_max_ (cm^−1^): 3078 (aromatic C-H), 2926 (aliphatic C-H asymmetric), 1668 (C=O), 1469, 1450, 1409 (C=N and C=C), 1197, 1072 (C-N), 829 (C-H out of plane deformation). ^1^H-NMR (400 MHz, δ ppm, DMSO-*d*_6_): 2.38 (3H, s, CH_3_), 3.22 (1H, dd, *J*_AX_ = 4.80 Hz, *J*_AB_ = 18.00 Hz, pyrazoline C_4_-H_A_), 3.93 (1H, dd, *J*_BX_ = 11.60 Hz, *J*_BA_ = 18.00 Hz, pyrazoline C_4_-H_B_), 4.60 (1H, d, *J* = 15.60 Hz, geminal proton, CO-C*H*), 4.73 (1H, d, *J* = 15.60 Hz, geminal proton, CO-C*H*), 5.61 (1H, dd, *J*_AX_ = 4.80 Hz, *J*_BX_ = 11.20 Hz, pyrazoline C_5_-H_X_), 7.15–7.18 (1H, m, thiophene C_4_-H), 7.26 (2H, d, *J* = 8.80 Hz), 7.33 (2H, d, *J* = 8.80 Hz), 7.36 (2H, d, *J* = 8.40 Hz), 7.49 (1H, m, thiophene C_3_-H), 7.77 (1H, d, *J* = 4.80 Hz, thiophene C_5_-H), 7.81 (2H, d, *J* = 8.00 Hz). ^13^C-NMR (100 MHz, DMSO-*d*_6_) δ (ppm): 21.80 (CH_3_), 36.09 (CH_2_, S-CH_2_), 43.41 (CH_2_, pyrazoline C_4_), 60.22 (CH, pyrazoline C_5_), 120.97 (C, aromatic), 127.00 (2CH, aromatic), 128.24 (2CH, aromatic), 128.81 (CH, aromatic), 129.29 (2CH, aromatic), 130.59 (2CH, aromatic), 130.93 (CH, aromatic), 131.82 (CH, aromatic), 132.69 (C, aromatic), 134.16 (C, aromatic), 140.98 (C, aromatic), 142.84 (C, aromatic), 152.29 (C, pyrazoline C_3_), 163.60 (C, aromatic), 164.17 (C, aromatic), 165.91 (C, C=O). Anal. Calcd. for C_24_H_19_ClN_4_O_2_S_2_: C, 58.23; H, 3.87; N, 11.32. Found: C, 58.19; H, 3.92; N, 11.25. MS (ESI) (*m*/*z*): 495 [M], 496 [M + H]^+^, 497 [M + H]^++^.

*1-[(5-(4-Methoxyphenyl)-1,3,4-oxadiazol-2-yl)thioacetyl]-3-(2-thienyl)-5-(4-chlorophenyl)-2-pyrazoline* (**12**). Yield: 72%; M.p. 146.8 °C. IR (KBr) ν_max_ (cm^−1^): 3082 (aromatic C-H), 2933 (aliphatic C-H asymmetric), 1670 (C=O), 1504, 1477, 1452, 1413 (C=N and C=C), 1251, 1178, 1026 (C-N), 840 (C-H out of plane deformation). ^1^H-NMR (400 MHz, δ ppm, DMSO-*d*_6_): 3.21 (1H, dd, *J*_AX_ = 4.80 Hz, *J_AB_* = 18.00 Hz, pyrazoline C_4_-H_A_), 3.84 (3H, s, OCH_3_), 3.92 (1H, dd, *J*_BX_ = 11.60 Hz, *J*_BA_ = 17.60 Hz, pyrazoline C_4_-H_B_), 4.57 (1H, d, *J* = 16.00 Hz, geminal proton, CO-C*H*), 4.71 (1H, d, *J* = 15.60 Hz, geminal proton, CO-C*H*), 5.61 (1H, dd, *J*_AX_ = 4.80 Hz, *J*_BX_ = 11.60 Hz, pyrazoline C_5_-H_X_), 7.08 (2H, d, *J* = 9.20 Hz), 7.16 (1H, *J* = 3.60 Hz, 4.80 Hz, thiophene C_4_-H), 7.25 (2H, d, *J* = 8.80 Hz), 7.34 (2H, d, *J* = 8.80 Hz), 7.50 (1H, dd, *J* = 1.20 Hz, 4.00 Hz, thiophene C_3_-H), 7.77 (1H, dd, *J* = 1.20 Hz, 4.80 Hz, thiophene C_5_-H), 7.86 (2H, d, *J* = 8.80 Hz). ^13^C-NMR (100 MHz, DMSO-*d*_6_) δ (ppm): 36.03 (CH_2_, S-CH_2_), 43.42 (CH_2_, pyrazoline C_4_), 56.23 (CH_3_, methoxy), 60.24 (CH, pyrazoline C_5_), 115.54 (2CH, aromatic), 116.10 (C, aromatic), 128.24 (2CH, aromatic), 128.80 (CH, aromatic), 128.92 (2CH, aromatic), 129.29 (2CH, aromatic), 130.89 (CH, aromatic), 131.74 (CH, aromatic), 132.70 (C, aromatic), 134.19 (C, aromatic), 140.98 (C, aromatic), 152.25 (C, pyrazoline C_3_), 162.77 (C, aromatic), 163.11 (C, aromatic), 164.26 (C, aromatic), 165.80 (C, C=O). Anal. Calcd. for C_24_H_19_ClN_4_O_3_S_2_: C, 56.41; H, 3.75; N, 10.96. Found: C, 56.39; H, 3.73; N, 10.98. MS (ESI) (*m*/*z*): 511 [M], 512 [M + H]^+^, 513 [M + H]^++^.

### 3.2. Biochemistry

#### 3.2.1. Cell Culture and Drug Treatment

U251 and U87 human glioblastoma cells were incubated in Dulbecco’s-modified Eagle’s medium (DMEM) (Wako Pure Chemical Industries, Osaka, Japan) supplemented with 10% fetal bovine serum (FBS) (Equitech-Bio, Kerrville, TX, USA). AsPC-1 human pancreas adenocarcinoma and Jurkat human leukemic T-cells were incubated RPMI 1640 (Wako Pure Chemical Industries), supplemented with 10% FBS. Peripheral blood mononuclear cells (PBMC) (Precision Bioservices, Frederick, MD, USA) were incubated RPMI 1640 and supplemented with 10% human serum AB (HS) (Gemini, Woodland, CA, USA). All media were supplemented with 89 µg/mL streptomycin (Meiji Seika Pharma, Tokyo, Japan) and cells were incubated at 37 °C in a humidified atmosphere of 95% air and 5% CO_2_. Growing cells were plated at 1 × 10^5^ cells/mL into 24-well microtiter tissue culture plates (Iwaki brand Asahi Glass Co., Chiba, Japan) and incubated for 24 h before the addition of the drugs (the optimal cell number for cytotoxicity assays was determined in preliminary experiments). Stock solutions (1 mM, 5 mM, 10 mM, 20 mM, and 50 mM) of compounds and cisplatin (Sigma-Aldrich, St. Louis, MO, USA) were prepared in dimethyl sulfoxide (DMSO; Wako Pure Chemical Industries) and in dimethylformamide (DMF; Wako Pure Chemical Industries) respectively, then were added to fresh culture medium. The concentration of DMSO and DMF in the final culture medium was 1%.

#### 3.2.2. MTT Assay for Cytotoxicity of Compounds

The level of cellular reduction of 3-(4,5-dimethylthiazol-2-yl)-2,5-diphenyltetrazolium bromide (MTT) (Dojindo Molecular Technologies, Kumamoto, Japan) was quantified as previously described in the literature with small modifications [38]. The tested compounds were incubated with cells to give a final concentration in the range 50–500 µM for 24 h. At the end of this period, MTT was added to cells in culture to give a final concentration of 0.275 mg/mL and incubated further for 4 h at 37 °C. The medium was removed and the formazan crystals were solubilized by addition of 100 µL DMSO to each well. After the solubilized crystals were diluted (1:10) with DMSO, 100 µL was transferred to wells of 96-well microtiter plates (Iwaki brand Asahi Glass Co.) and the absorbance at 550 nm was measured using a microplate spectrophotometer Infinitive M1000 (Tecan, Groding, Austria). Every concentration was repeated in three wells and IC_50_ values were defined as the drug concentrations which reduced absorbance to 50 % of control values.

#### 3.2.3. Detection of Apoptotic and Necrotic Cells

U251 cells were incubated with compound **11** at IC_50_ concentration for 3 h and 24 h. Then, apoptotic/necrotic/healthy cells detection kit protocol was applied according to the manufacturer’s instruction manual (PromoKine, Heidelberg, Germany) [39,40]. After the cells were washed twice with 1 × binding buffer, a staining solution containing 50 µL of 1 × binding buffer, 2 µL of FTIC-Annexin V solution, 2 µL of ethidium homodimer III solution and 2 µL of Hoechst 33342 solution was added and the cells were incubated for 15 min at RT, protected from light. Cells were washed with 1 × binding buffer and analyzed by all-in-one fluorescence microscope Biorevo Fluorescence BZ-9000 (Keyence, Osaka, Japan).

#### 3.2.4. DNA Cleavage Assay

The DNA cleavage activities of the compounds were studied using supercoiled pUC19 DNA and analyzed by agarose (Takara, Kyoto, Japan) gel electrophoresis Mupid-2x (Mupid, Tokyo, Japan). pUC19 DNA (2 µg) was treated with compounds in water and Tris/boric acid (Nacalai Tesque, Kyoto, Japan) buffer (10 mM, pH 8.5) in the presence and absence of iron (II) sulfate heptahydrate (FeSO_4_·7H_2_O; 30 µM) (Wako Pure Chemical Industries), hydrogen peroxide (H_2_O_2_; 30 µM) (Tokyo Chemical Industry Co., Tokyo, Japan) and ascorbic acid (30 µM) (Tokyo Chemical Industry Co.) as an activator. The reaction mixture was incubated at 37 °C for 1.5 h before the addition of EDTA (Dojindo Molecular Technologies) and loading buffer (Takara, Kyoto, Japan). Agarose gel electrophoresis of pUC 19 DNA was performed at 100 V for 40 min in 1% slab gels containing ethidium bromide (Wako Pure Chemical Industries) in Tris/Boronic acid/EDTA buffer. DNA was visualized by photographing the fluorescence of intercalated ethidium bromide under a UV illuminator (Nippon Genetics Co., Tokyo, Japan).

## 4. Conclusions

In the present paper, new pyrazoline derivatives were synthesized and investigated for their antiproliferative effects on AsPC-1 human pancreatic adenocarcinoma, U87, and U251 human glioblastoma cell lines. Detailed investigation of compound **11** against AsPC-1, U87, U251 cell lines and tumor selectivity of this compound on blood cells (PBMC and Jurkat cells) were also carried out. Among these compounds, compound **11** was the most effective anticancer agent against AsPC-1 and U251 cancer cell lines and exhibited significant tumor selectivity. Therefore, compound **11** was chosen for apoptosis/necrosis evaluation and DNA-cleavage analysis in U251 cells. Compound **11**-treated U251 cells showed apoptotic activity at low concentration (1.5 µM). Interestingly, DNA-cleaving efficiency of this ligand was more significant than cisplatin. This outcome pointed out the relationship between the DNA cleavage and the cell death.

In the view of this study, further research can be carried out on the development of new effective anticancer agents by the modification of compound **11**.
